# Serglycin as a potential biomarker for glioma: association of serglycin expression, extent of mast cell recruitment and glioblastoma progression

**DOI:** 10.18632/oncotarget.15820

**Published:** 2017-03-01

**Authors:** Ananya Roy, Sanaz Attarha, Holger Weishaupt, Per-Henrik Edqvist, Fredrik J. Swartling, Michael Bergqvist, Florian A. Siebzehnrubl, Anja Smits, Fredrik Pontén, Elena Tchougounova

**Affiliations:** ^1^ Uppsala University, Department of Immunology, Genetics and Pathology, Rudbeck Laboratory, Uppsala, Sweden; ^2^ Science for Life Laboratory, Uppsala University, Uppsala, Sweden; ^3^ Umeå University, Department of Radiation Sciences, Umeå, Sweden; ^4^ Cardiff University School of Biosciences, European Cancer Stem Cell Research Institute, Cardiff, United Kingdom; ^5^ Uppsala University, Department of Neuroscience, Neurology, Uppsala, Sweden; ^6^ Swedish University of Agricultural Sciences, Department of Biomedical Sciences and Veterinary Public Health, Uppsala, Sweden; ^7^ Institute of Neuroscience and Physiology, Department of Clinical Neuroscience, Sahlgrenska Academy, Gothenburg University, Gothenburg, Sweden

**Keywords:** mast cell, glioma, serglycin, CD44, ZEB1

## Abstract

Serglycin is an intracellular proteoglycan with a unique ability to adopt highly divergent structures by glycosylation with variable types of glycosaminoglycans (GAGs) when expressed by different cell types. Serglycin is overexpressed in aggressive cancers suggesting its protumorigenic role. In this study, we explored the expression of serglycin in human glioma and its correlation with survival and immune cell infiltration. We demonstrate that serglycin is expressed in glioma and that increased expression predicts poor survival of patients. Analysis of serglycin expression in a large cohort of low- and high-grade human glioma samples reveals that its expression is grade dependent and is positively correlated with mast cell (MC) infiltration. Moreover, serglycin expression in patient-derived glioma cells is significantly increased upon MC co-culture. This is also accompanied by increased expression of CXCL12, CXCL10, as well as markers of cancer progression, including CD44, ZEB1 and vimentin.

In conclusion, these findings indicate the importance of infiltrating MCs in glioma by modulating signaling cascades involving serglycin, CD44 and ZEB1. The present investigation reveals serglycin as a potential prognostic marker for glioma and demonstrates an association with the extent of MC recruitment and glioma progression, uncovering potential future therapeutic opportunities for patients.

## INTRODUCTION

Glioblastoma (GBM) is one of the most lethal human cancers. The majority (∼90%) of these tumors are classified as primary GBMs that develop rapidly in patients without any clinical or histologic evidence of a less malignant precursor lesion. A small population of younger patients however show incidence of secondary GBMs. Even though these two GBM classes display similar histologic appearance, primary and secondary GBMs have been shown to be distinct tumor entities [1]. There is no early detection for primary GBM. The widespread tumor cell infiltration makes complete surgical excision impossible without damaging vital or eloquent structures in the brain. Despite the advances in radiation and chemotherapy and the efforts to design new treatment strategies, GBMs invariably recur as aggressive, therapy-resistant relapses and patients rapidly succumb to these tumors. The prognosis for primary GBMs remains among the poorest, with a median survival for patients of just over one year [2].

The essential role of tumor microenvironment (TME) in cancer progression has been well documented and recent findings indicate that the microenvironmental components contribute to the proliferative and migratory potential of cancer cells and represent promising therapeutic targets and diagnostic markers [3]. For instance, the evasiveness of glioma from immune responses is considered to involve chronic inflammation and recruitment of myeloid suppressor cells, microglia and T-regulatory cells that effectively obstruct an anti-tumor immune response [4]. Nonetheless, the interrelation between the immune system and glioma remains incompletely understood.

Mast cells (MCs) are key regulators of the TME, affecting angiogenic processes, immune modulation and tissue remodeling [5]. The uniqueness of MCs lies in their functional plasticity [6]. The life and activity of MCs as well as the magnitude and nature of their responses to stimulation is regulated and fine-tuned by various environmental and genetic factors. The role of MCs in cancer is still controversial since depending on the type and malignancy grade of the cancer it can be either supportive or detrimental for progression and establishment for malignant tumors [7]. We previously reported that gliomas also contain MCs and we addressed the role for different axes in chemotactic infiltration of MCs into glioma [8–10].

The GBM microenvironment contains a complex mixture of various cell types, extracellular matrix (ECM) proteins and glycosaminoglycans (GAGs). Serglycin proteoglycan is the dominant proteoglycan species in most hematopoietic cells including MCs and it is also expressed in non-hematopoietic cells like endothelial cells, fibroblasts and certain aggressive cancer cells [11]. In its intracellular form its function is related to packaging of mediators in storage granules and secretory vesicles. Upon secretion serglycin can modulate the activities of binding partners by either/or protecting, transporting, activating and interacting with target cells or mediators in the ECM [11]. Serglycin is the major intracellular proteoglycan species in MCs, where it is localized in the granules and implicated in the storage of proteases, whereas upon activation it is secreted into the extracellular matrix along with the proteases. Several studies demonstrated that serglycin with different GAG chains and sulfation pattern is expressed in numerous human hematopoietic and non-hematopoietic tumors [12–14]. Recently it was also reported that serglycin expression is essential for metastatic growth and dissemination [11]. The role of serglycin in malignancies is rather intriguing because it appears to mediate interactions between tumor cells and TME, suggesting for serglycin to be a modulator within the complex tumor “ecosystem” [15, 16].

Here we demonstrate that serglycin is expressed in glioma and predicts poor survival of patients. Serglycin expression is positively correlated with MC accumulation in tissue microarrays (TMAs) of a large cohort of human high-grade glioma tissues. We show that serglycin can promote aggressiveness in glioma by increasing the expression of ZEB-1 and vimentin. In addition, the expression of serglycin and ZEB-1 is positively correlated in high-grade glioma. Further, we provide evidence that MCs can have a potential role in serglycin induced tumor progression by activating a CD44-related signaling pathway.

The present investigation reveals serglycin as a potential prognostic marker for glioma. It also demonstrates the association with the extent of MC recruitment and glioma progression, suggesting a functional link between serglycin, MCs and glioma malignancy grade.

## RESULTS

### Serglycin expression in human cancers. High serglycin expression in human primary glioblastoma correlates with low survival rate

Serglycin has been reported to be overexpressed in human cancers [14, 17, 18]. In order to get an overview of the extent of SRGN expression in various cancers and compare it to GBM patients, we analyzed a large set of publicly available expression data in the R2 analysis webtool (R2 Genomics and Visualization Platform, Oncogenomics, AMC) to explore the landscape of SRGN expression patterns across major human cancers (prostate, ovary, lung, kidney, endometrium, colon, breast, myeloma, hepatocellular carcinoma (HCC), glioblastoma (GBM), non-small cell lung carcinoma (NSCLC), medulloblastoma (MB), B-cell lymphoma). Indeed we found SRGN expression to be high in these cancers with a correlation to malignancy grade.

**Figure 1:**
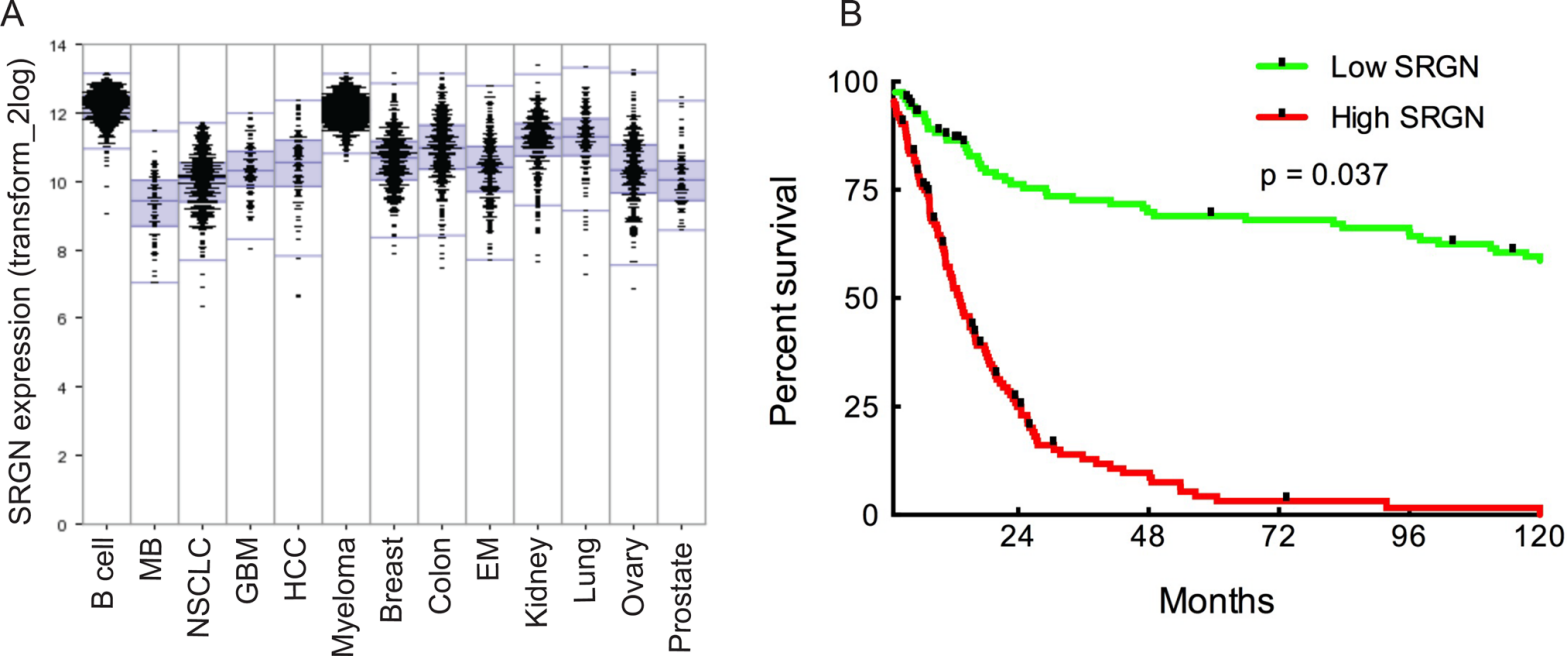
SRGN is overexpressed in human cancers High SRGN expression in human glioma correlates with low survival rate. (**A**) Comparison of SRGN expression patterns across major human cancers: prostate (*n* = 72), ovary (*n* = 256), lung (*n* = 121), kidney (*n* = 261), endometrium (*n* = 209), colon (*n* = 315), breast (*n* = 351), myeloma (*n* = 542), hepatocellular carcinoma (HCC) (*n* = 91), glioblastoma (GBM) (*n* = 84), non-small cell lung carcinoma (NSCLC) (*n* = 410), medulloblastoma (MB) (*n* = 73), B-cell lymphoma (*n* = 420). All clinical data for this analysis was obtained from the R2 genomic analysis platform. (**B**) Survival analysis comparing GBM patient survival with high and low SRGN expression. Log-rank *p*-value = 0.037 (*n* = 504). Clinical data for the TCGA patients was obtained from the TCGA portal and in each case the maximum value of the survival time available was used. Significant differences in survival between groups were evaluated using a Kaplan-Meier analysis with censoring at confidence interval 95%.

The analysis also demonstrated high SRGN expression in GBM (Figure 1A). In order to assess the potential relation of SRGN signaling in human GBM we analyzed the TCGA dataset of 540 GBM patient samples and found an upregulation of SRGN in these patients. We used the well-established markers EGFR and PTEN as positive and negative controls for upregulated SRGN expression in human GBM. To evaluate a potential correlation between the SRGN expression and patient survival we analyzed the TCGA GBM dataset for 540 patients with matching transcriptome and survival data. First, we sorted the TCGA patients by SRGN expression from high to low and performed a Kaplan-Meier survival analysis to compare the top 25% patients (with high expression) and the bottom 25% of patients (with low expression). This analysis showed a significant difference in survival between the two groups (Figure 1B, log-rank *p*-value = 0.037) with high SRGN expression giving a poor prognosis.

Furthermore, a closer statistical analysis revealed varied expression levels of SRGN among the different GBM subtypes (Supplementary Figure 1). Patient-derived glioma cell cultures used in this study are part of the Uppsala University Human Glioma Cell Culture (HGCC) collection that comprises well characterized GBM-derived cell cultures [19]. A survival analysis based on the HGCC data (*n* = 40), although not significant, showed a similar tendency of high SRGN expression being associated with poor prognosis (data not shown).

### The level of serglycin expression is correlated with human glioma malignancy grade. MCs as potential modulators of serglycin expression in GBM

**Figure 2:**
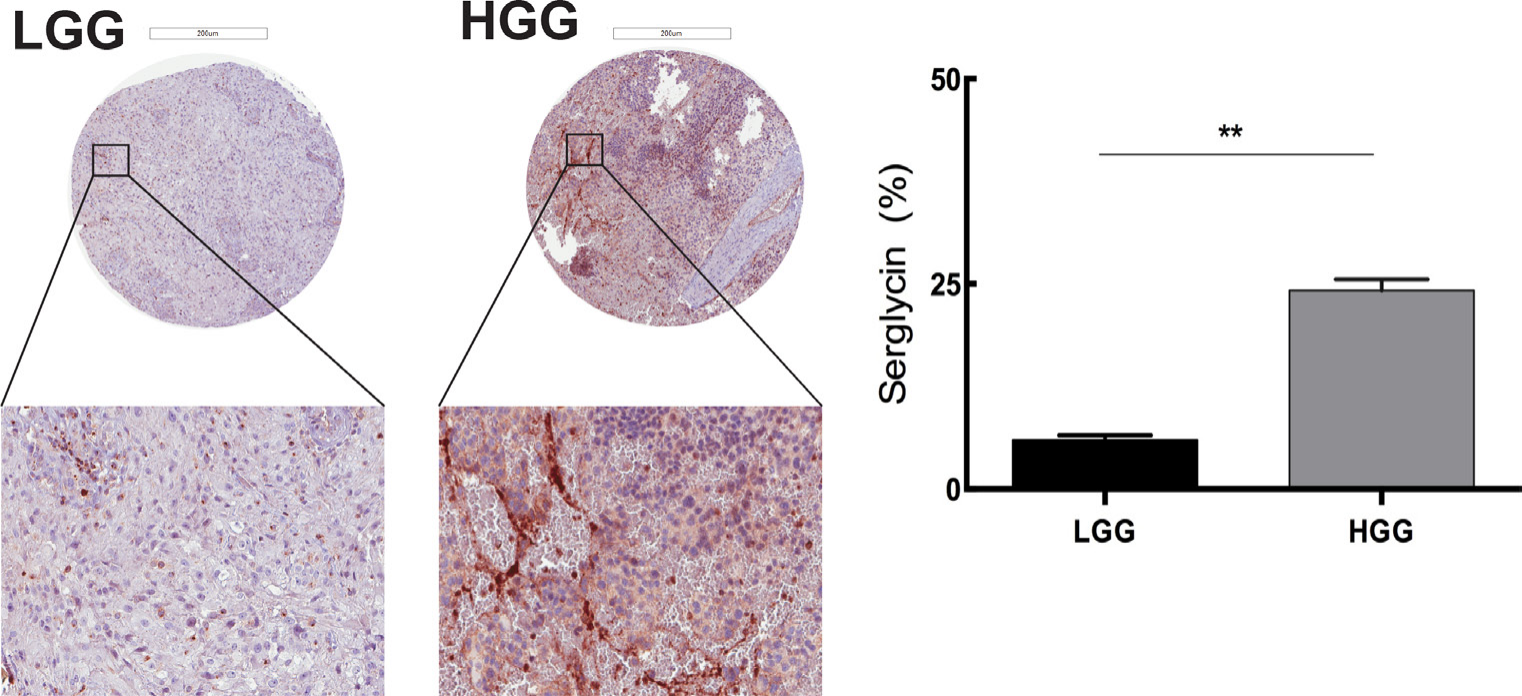
Serglycin expression is correlated with human glioma malignancy grade Representative TMA cores of low-grade glioma (LGG) (upper left panel) and high-grade glioma (HGG) (upper right panel) for serglycin expression. Selected areas from upper panels are magnified in the middle panel. TMAs involved tumor tissue from high-grade gliomas (anaplastic gliomas and glioblastomas, *n* = 101) and gliomas WHO grade II (astrocytomas, oligoastrocytomas and oligodendrogliomas grade II, *n* = 87). Scale bar = 200 μm. Quantification of the SRGN expression levels (lower panel) shows significantly higher level of SRGN expression in the high-grade glioma TMA. Data is expressed as mean + SEM values and significance differences are indicated in the figure. **p < 0.01.

Although there are studies investigating proteoglycans as therapeutic targets in the brain TME [20] there is no population-based study of tissue morphology and serglycin expression in glioma. We performed tissue analysis of serglycin expression in human low-grade (astrocytomas, oligoastrocytomas and oligodendrogliomas grade II, *n* = 87) and high-grade glioma (anaplastic gliomas and glioblastomas, *n* = 101) TMAs. High-grade glioma demonstrated significantly higher expression of serglycin in comparison to low-grade glioma (Figure 2).

**Figure 3:**
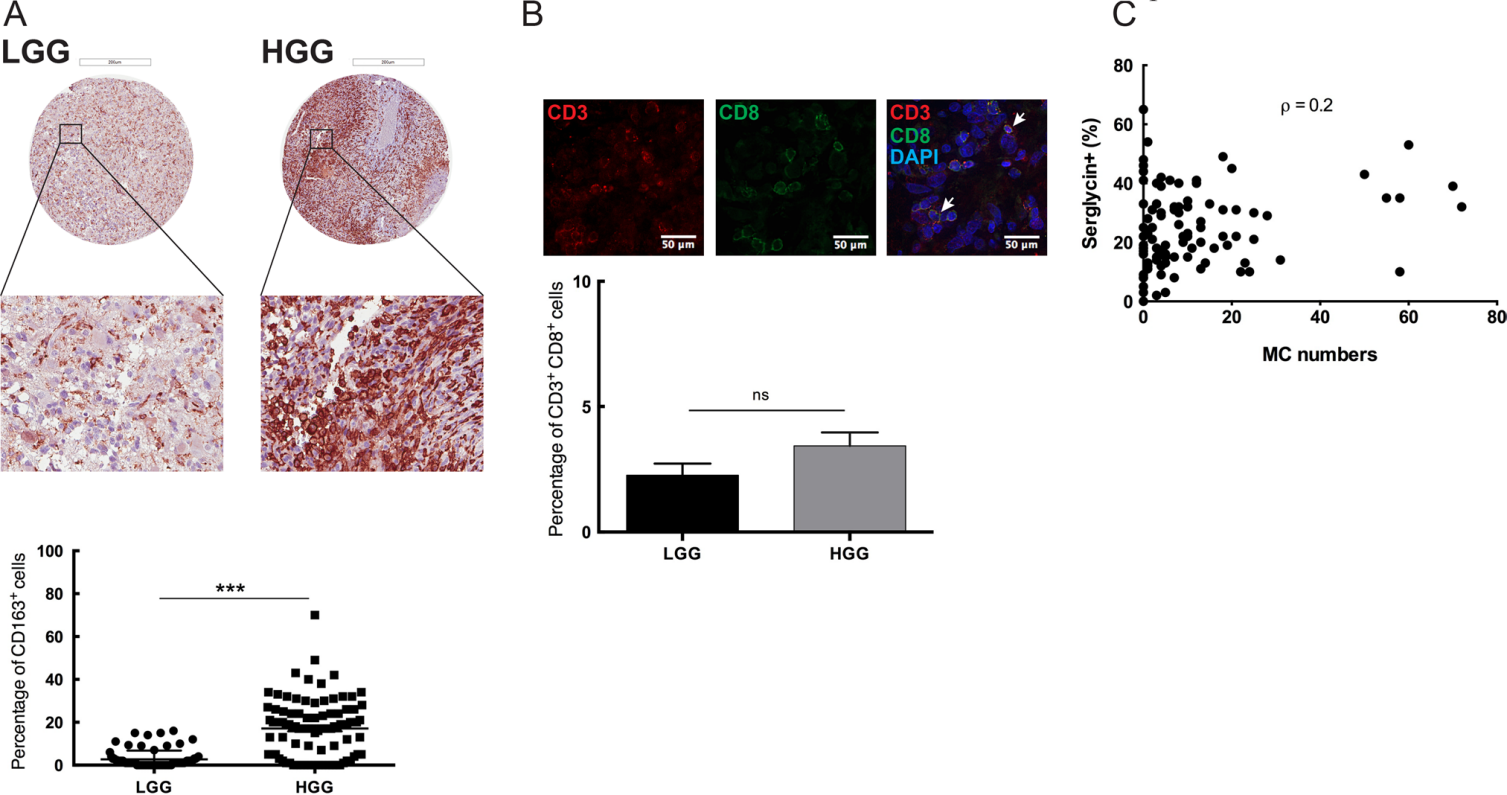
Immune cell infiltration in the glioma microenvironment: MCs as potential modulators of SRGN expression in GBM (**A**) Immunohistochemical staining for CD163 in human low- and high-grade glioma TMAs. Representative TMA cores of low-grade glioma (upper left panel) and high-grade glioma (upper right panel) for CD163 expression. Selected areas from upper panels are magnified in the middle panel. TMAs included tumor tissue from high-grade gliomas (anaplastic gliomas and glioblastomas, *n* = 101) and gliomas WHO grade II (astrocytomas, oligoastrocytomas and oligodendrogliomas grade II, *n* = 87). Quantification of the CD163 expression levels (lower panel) shows significantly higher CD163 expression in the high-grade glioma TMA compared to the low-grade glioma. ***p < 0.001 (**B**) Immunofluorescence staining for CD3 and CD8 in human glioma TMAs. Representative staining's for CD3 (upper left panel), CD8 (upper middle panel) and CD3CD8 with DAPI staining (upper right panel). TMAs included tumor tissue from high-grade gliomas (anaplastic gliomas and glioblastomas, *n* = 101) and gliomas WHO grade II (astrocytomas, oligoastrocytomas and oligodendrogliomas grade II, *n* = 87). Quantification of the CD3 and CD8 expression levels (lower panel) shows no significant difference in the CD3+CD8+ cells between the low- and high-grade glioma TMA. Data is expressed as mean values + SEM and significance differences are indicated in the figure. ns = not significant. Scale bar = 200 μm (**C**) Spearman's rank correlation analysis between the number of infiltrating MCs and serglycin expression level, (Spearman's rho (ρ) = 0.2). LGG = low-grade glioma, HGG = high-grade glioma.

The glioma microenvironment has been shown to contain varying levels of immune cell infiltration, e.g. of MCs, macrophages and T cells [21]. Most of these immune cells have serglycin repertoire and MCs have a high capacity for serglycin expression as compared with the other cell types [22]. To map the immune cell infiltration in low- and high-grade gliomas and to investigate potential correlation to serglycin expression levels we performed immunohistochemistry for CD163^+^ tumor-associated macrophages (TAMs), MCs (tryptase positive) and tumor-infiltrating CD3^+^ and CD8^+^ lymphocytes (TILs) (Figure 3). Previous studies from our lab have shown that the number of MCs in human glioma is dependent on malignancy grade [9] but their functional contribution to tumor progression remains unclear. In the present investigation we also found that CD163^+^ cell numbers were significantly higher in the high-grade glioma TMAs as compared to the low-grade (Figure 3A). Infiltrating CD3^+^ and CD8^+^ T cells were very scarce in both low- and high-grade glioma tissues and were not abundant as other infiltrating cells (Figure 3B). This observation is in line with some studies showing that decreased CD3^+^ and CD8^+^ T cells in advanced glioma is associated with profound host immunosuppression [23], whereas others attributed these low numbers to the blood brain barrier (BBB) [24].

Statistical analysis of the high-grade glioma TMAs for serglycin expression and MC numbers revealed a positive correlation (Spearman's rho (ρ) = 0.2) between the number of infiltrating MCs and the relative intensity of staining for serglycin (Figure 3C), suggesting that MCs can be considered as one of the modulator of serglycin expression in GBM.

### Co-culture of MCs and glioma cells induces the expression of SRGN and CD44 in glioma cells and is accompanied by increased expression of a distinct set of proinflammatory cytokines in both cell types

Considering the positive correlation between MC number and serglycin expression in TMAs and our previous studies on MC recruitment to glioma [8–10], we postulate that serglycin expression modulated by MCs can support glioma progression. In order to study the crosstalk between MCs and glioma cells, individual patient-derived and well-characterized HGCC cell lines were grown in co-culture with LAD2 cells.

Considering the variety of receptors and mediators expressed by MCs, we were interested in studying the bidirectional/mutual induction of mediators between MCs and glioma cells.

**Figure 4:**
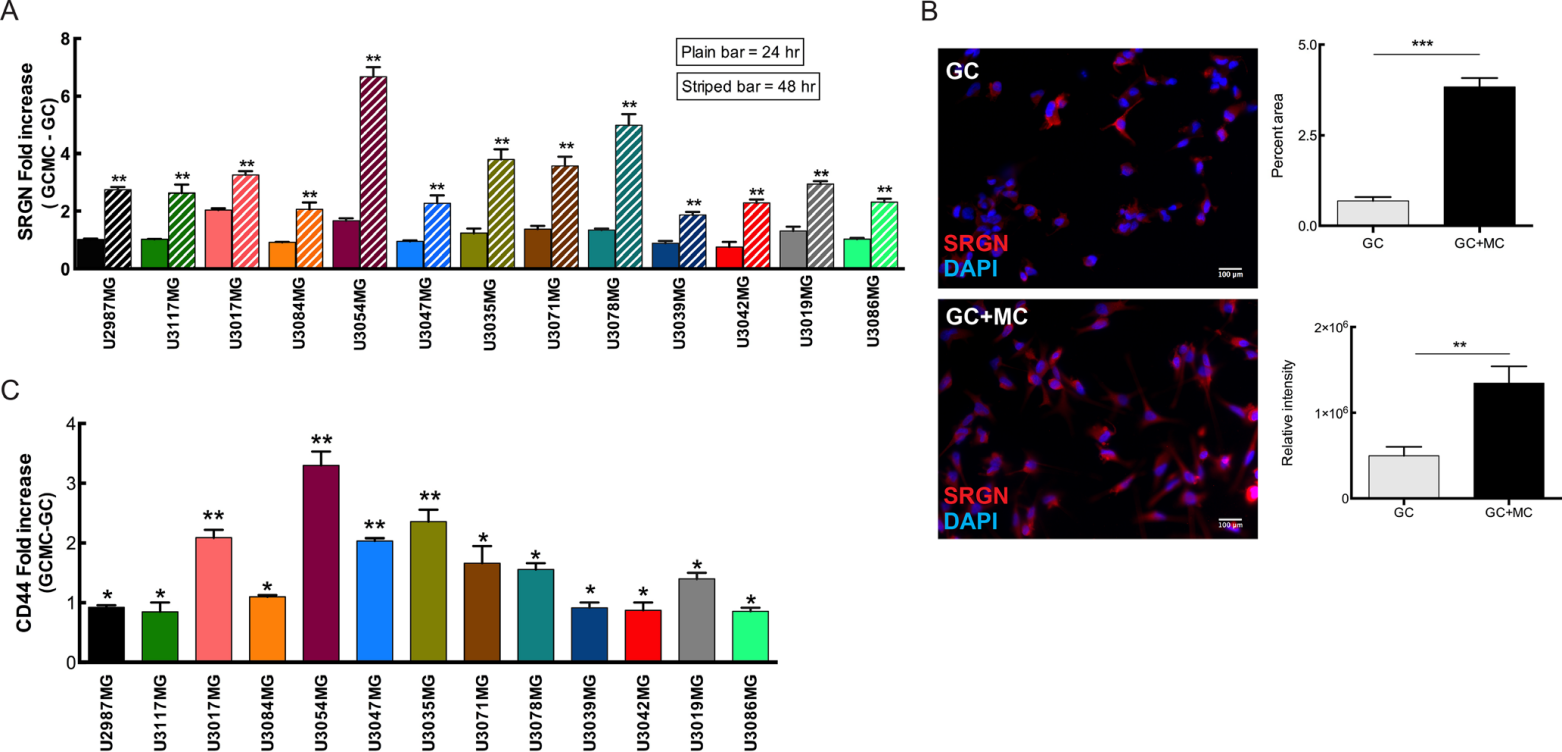
MCs can modulate SRGN and CD44 expression in glioma cells upon co-culture (**A**) Expression analysis of SRGN in the glioma cells after co-culture with LAD2 cells for 24 hours (plain bars) and 48 hours (striped bars) show a significant increase in SRGN expression levels at 48 hours. (**B**) Left panel shows representative pictures of serglycin staining in glioma cells. Left upper panel: control glioma cells and left bottom panel: glioma cells after 48 hours co-culture with LAD2 cells. Right panel shows the quantification of staining. Right upper panel: percentage area of serglycin expression, right bottom panel: relative density of serglycin staining (**C**) qPCR analysis for the expression of CD44 in the glioma cells after co-culture with LAD2 cells for 48 hours. Scale bar = 100 μm All the results are represented as increase in fold expression compared to the expression levels without co-culture. The experiments were performed 3 times, with triplicates in each case. Data is expressed as mean values + SEM and significance differences are indicated in the figure. GC = glioma cells, GC+MC = glioma cells co-cultured with LAD2 cells. *p < 0.05, **p < 0.01, ***p < 0.001 versus cells grown without co-culture.

Analysis of the glioma cell lines after glioma cell-mast cell (GC-MC) co-culture at different time-points indicated an increased SRGN (Figure 4A–4B) and CD44 expression (Figure 4C) as compared when culturing glioma cells in normal cell culture media. CD44, a transmembrane glycoprotein that is involved in cell–cell and cell-matrix interactions either through its affinity for serglycin, hyaluronic acid (HA), or other ECM components, has been shown to be upregulated in various cancer types including glioma and increased expression of CD44 promotes the metastasizing and migratory potential of tumor cells [25]. We postulate that increased expression of serglycin, as a main interacting partner for CD44, upon co-culture with MCs induces the CD44 expression on glioma cells to initiate a bidirectional signaling between CD44 and its surroundings. Additionally, serglycin can also initiate an autocrine signaling pathway to enhance the proliferation and metastasing capacity of the tumor cells [26]. Moreover, potential crosstalk between serglycin and CD44, may function as an amplifier modulating a variety of tumor induction signals [25].

**Figure 5:**
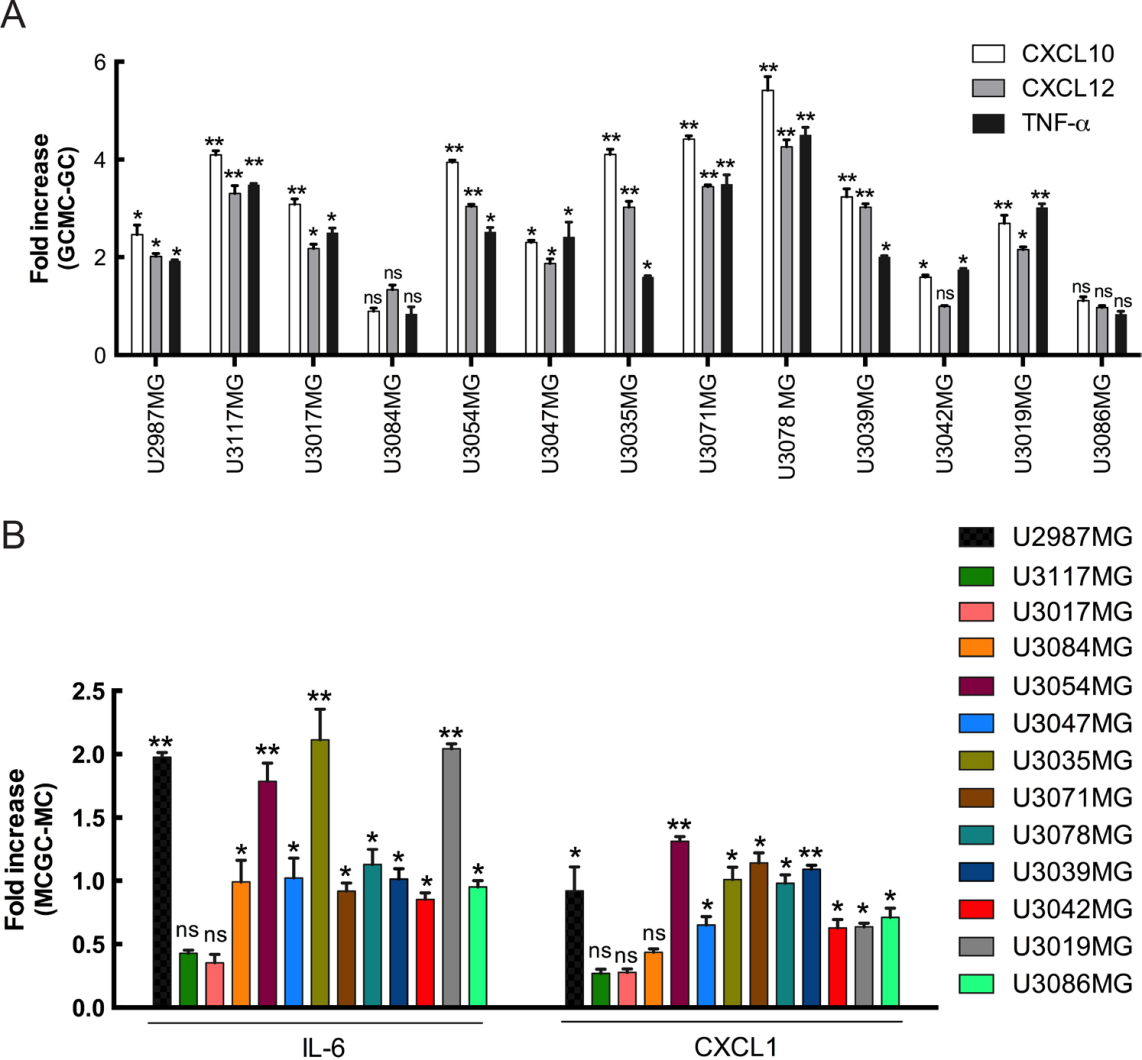
Increased SRGN expression is accompanied by increased expression of a distinct set of mediators in both glioma cells and MCs (**A**) qPCR analysis for the expression of CXCL10, CXCL12 and TNF-α in the glioma cells after co-culture with LAD2 cells for 48 hours show a significant increase in expression in the co-cultured glioma cells as compared to not co-culturing with LAD2 cells. (**B**) qPCR analysis for the expression of CXCL1 and IL-6 in the LAD2 cells after co-culture with the glioma cells for 48 hours show a significant increase in expression in the co-cultured LAD2 cells as compared to not co-culturing with glioma cells. All the results are represented as increase in fold expression compared to the expression levels without co-culture. The experiments were performed 3 times, with triplicates in each case. Data is expressed as mean values + SEM and significance differences are indicated in the figure. GC = glioma cells, MC = LAD2 cells, GCMC = glioma cells co-cultured with LAD2 cells, MCGC = LAD2 cells co-cultured with glioma cells. ns = not significant, *p < 0.05, **p < 0.01 versus cells grown without co-culture.

Chemokines and their receptors serve as critical communication bridges between tumor cells and stromal cells to create a microenvironment beneficial for tumor development. Their aberrant overexpression in various cancers strongly promotes growth and proliferation through multiple signaling pathways. A link between MCs and the CXCL12 in glioma TME had previously been established. In our study, we observe a marked increase in the expression of CXCL10 and CXCL12 in the glioma cells after co-culture as compared to normal culturing of glioma cells (Figure 5A). CXCL12 has been reported to be overexpressed in various cancer types which, in turn, strongly promotes proliferation, migration and invasion [27]. CXCL12 is also a potent chemotactic agent for lymphocytes [28, 29] and can induce an increase in monocyte-macrophage infiltration into the glioma microenvironment [30] MC infiltration in the glioma TME leads invariably to a tissue inflammation. Analysis of proinflammatory cytokines showed an increase in TNF-α expression in the MC co-cultured glioma cells (Figure 5A).

Interestingly, an upregulation of IL-6 and CXCL1 mRNA in LAD2 cells was observed in response to the co-culture (Figure 5B). Both cytokines have been associated with tumor related inflammation and disease progression [31, 32].

### Serglycin can induce aggressiveness and propagation in glioma cells

Our finding suggests that glioma cells induce expression of specific MC mediators to assist them in propagation and establishment of a suitable TME.

**Figure 6:**
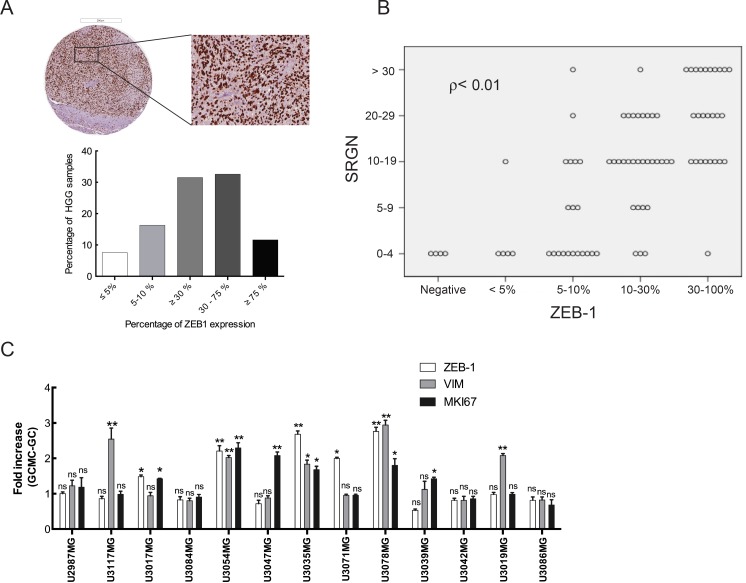
Serglycin can induce aggressiveness and propagation in glioma cells (**A**) Immunohistochemical staining for ZEB-1 in high-grade human glioma TMAs. Representative high-grade glioma TMA core (upper left panel) and selected magnified area (upper right panel) showing ZEB-1 expression and quantification of the ZEB-1 expression level (lower panel). HGG TMAs included tumor tissue from high-grade gliomas (anaplastic gliomas and glioblastomas, *n* = 101). Scale bar = 200 μm. (**B**) ZEB-1 expression levels positively correlates to the serglycin expression levels in high-grade glioma (Pearson correlation ρ < 0.01). (**C**) Expression analysis for ZEB-1, vimentin and Ki67 in the glioma cells after co-culture with LAD2 cells for 48 hours. All the results are represented as increase in fold expression compared to the expression levels without co-culture. The experiments were performed 3 times, with triplicates in each case. Data is expressed as mean values + SEM and significance differences between GC+MC and GC are indicated in the figure. *p < 0.05, **p < 0.01 versus cells grown without co-culture. LGG = low-grade glioma, HGG = high-grade glioma. GC = glioma cells, GC+MC = glioma cells co-cultured with LAD2 cells.

Considering the abundance of serglycin in high-grade gliomas and increased serglycin expression in glioma cells after GC-MC co-culture, we decided to further analyze the high-grade glioma TMAs for potential correlation between serglycin expression and markers related to tumor aggressiveness. One such marker that was induced in our co-cultures, CD44, is widely expressed in glioma, and showed strong and widespread cytoplasmic staining in immunohistochemistry (data not shown). However, the extent of intense and diffuse staining rendered it unsuitable for quantification. As CD44 is associated with glioma stem cells [33] and recent studies indicate a reciprocal link between CD44 and ZEB1 [34] we tested whether ZEB1 may be a substitute marker for CD44. ZEB1 is associated with malignancy in glioma [35] and analysis of GBM patient specimens demonstrated that proinflammatory cytokines, such as CXCL12, IGFBP2, IL-1ß, TNF-a and MIF, correlate significantly with ZEB1 expression in tumor specimens (Supplementary Figure 2). Furthermore, our previous studies have shown CXCL12 and MIF secreted from glioma cells to be potent chemoattractants for MC recruitment in glioma [8, 9]. Indeed, high-grade glioma TMAs immunostaining for ZEB1 revealed abundant expression of this protein (Figure 6A), as well as a positive correlation between ZEB1 and serglycin levels (*ρ* < 0.01, Figure 6B). We further analyzed expression of genes involved in glioma progression, to investigate the effect of increased serglycin expression in the glioma cells upon GC-MC co-culture. As seen in Figure 6C and Supplementary Figure 3, the expression of ZEB1 and vimentin as well as Ki-67 (MKI67) as proliferation marker, was increased in co-cultured glioma cells as compared to glioma cells cultured without LAD2 cells. This data suggests that serglycin can support and further induce progression and propagation of glioma cells.

## DISCUSSION

Despite recent therapeutic advances, GBMs remain devastating tumors and there is a continuing need for improvement in identification and characterization of potential prognostic biomarkers. The glioma microenvironment is a network of complex interactions between diverse cell types, including immune cells, fibroblasts, mesenchymal stem cells and endothelia. The microenvironment also includes the ECM and the factors produced by the host cells as well as the tumor cells. A balanced and normal ECM, comprising of proteins, glycoproteins and proteoglycans, is important to prevent tumor progression and tumor angiogenesis. Hence, recent studies have focused largely on evaluating the role of this microenvironment in tumor formation and progression [21].

Recent studies have shown overexpression of serglycin in several aggressive cancer types, including breast cancer, hepatocellular carcinoma, nasopharengeal carcinoma and myeloma. Although several recent studies have demonstrated the emerging role of serglycin in tumorigenesis [15, 18, 36] not much information is available on the expression and distribution of this proteoglycans in brain tumors. By performing bioinformatic analysis for SRGN expression from large patient datasets we show significantly elevated SRGN levels in various malignant cancers, including GBM. The analysis of the TCGA GBM dataset also revealed significant upregulation of SRGN. Kaplan-Meier survival curves using the TCGA GBM dataset show that high SRGN expression results in poor prognosis for GBM patients. Histological analysis in our study reveals that serglycin is expressed in human glioma and the level of expression is increasing with glioma grade.

We previously demonstrated that recruitment of MCs in glioma is dependent on several glioma-secreted factors [6–8]. We also found that MC recruitment increases with tumor grade. Serglycin is the major proteoglycan in MCs and can act as an immune modulator in the TME and enhance the resistance of tumor cells to various therapeutic agents. The presence of serglycin is associated with increased invasion and metastasis of tumor cells. Furthermore, its charged GAG moiety makes it an ideal binding partner for molecular effectors like proteolytic enzymes, chemokines, cytokines and growth factors in regulation of their biosynthesis, secretion and activity by protecting and accompanying them to specific target sites [16]. We examined the infiltration of immune cells, particularly CD163^+^ TAMs, MCs, CD3^+^ and CD8^+^ T cells, and found significant infiltration of TAMs and MCs to high-grade gliomas whereas CD3^+^ and CD8^+^ T cell infiltration was selective and not widespread. We found a positive correlation between MC numbers and serglycin expression in human high-grade glioma tissues implying MCs as a major serglycin source.

Studies report significantly elevated SRGN expression in breast and lung cancer cell lines, as compared to colon and gastric cancer cell lines [36]. We analyzed the constitutive SRGN expression levels in 40 primary HGCC cell lines and found moderate but variable expression levels. To evaluate the effect of MCs on glioma cells and their combined effect on the ECM, co-culture analysis was performed. We found that SRGN is overexpressed in GBM cells upon interaction with MCs and that secreted serglycin can thereby stimulate aggressive phenotypes of the glioma cells through autocrine signaling pathways [26]. Also serglycin both from the glioma cells and MCs, via its interaction with CD44, can enhance growth, survival and differentiation of the cancer cells. CD44 is a versatile multifunctional cell surface glycoprotein involved in a variety of biological properties and physiological activities of cells. Its binding mechanism has been mostly noted in context to HA and the associated GAGs. Serglycin has been reported as a novel ligand for CD44 [37], and to the best of our knowledge is the only likely candidate of all MC mediators that can directly bind to CD44.

MCs express a plethora of various receptors e.g. members of the CXCL10/CXCR3 and CXCL12/CXCR4 family, which upon binding to its ligand can activate MCs [38]. Expression analysis of cytokines secreted by glioma cells co-cultured with MCs, revealed a significant increase in expression of CXCL10, CXCL12 and TNF-α. CXCL10 induces a wide spectrum of physiological and pathological effects including chemotaxis, induction of apoptosis, regulation of cell growth and mediation of angiostatic effects via the CXCR3 receptor, resulting in immune dysfunction, tumor development, metastasis and dissemination. Furthermore, the CXCL12/CXCR4/CXCR7 axis has been implemented in carcinogenesis and tumor progression [27]. TNF-α, which has been shown to be involved in malignant diseases [39], had increased expression in the co-cultured glioma cells, which in turn can bind to its receptor on the MCs to activate them, thereby expanding the inflammatory network and facilitating proper establishment of the malignancy.

We also observed an increase in IL-6 and CXCL1 expression in MCs co-cultured with glioma cells. IL-6 secreted in the TME has been suggested to promote development and progression of certain cancers by driving progression and EMT [32]. Dysregulated expression of CXCL1 and IL-6 have also been attributed to enhanced angiogenesis and tumorigenesis in cancer cells via constitutive activation of NF-κB [40]. Moreover, several cytokines and growth factors including CXCL1 and IL-6 have been shown to exert their effect via their ability to bind to serglycin [15]. In this case serglycin acts as the modulator supplying an ECM microenvironment platform for exerting eventual effects.

Investigation of markers related to cancer progression in glioma cells co-cultured with MCs show a significant induction of ZEB1 and vimentin accompanied by a positive induction of glioma cell proliferation in co-culture with MCs. An increase in ZEB1 expression was also detected in human high-grade glioma TMAs. Statistical analysis of the expression of serglycin and ZEB1 in high-grade glioma showed a positive correlation. Our previous studies demonstrated a positive correlation between MC infiltration and the grade of glioma. Hence we believe that MC infiltration in high-grade glioma tissue can induce the aggressiveness of the glioma cells even further presumably as orchestrated by serglycin.

In conclusion, we showed elevated expression of serglycin in a large cohort of human high-grade glioma TMAs and demonstrated a correlation between glioma grade and serglycin expression level. We identified a positive correlation between serglycin expression and MC numbers. In line with these findings, *in vitro* analysis of patient-derived GBM cell lines co-cultured with MCs showed an induction of serglycin expression in glioma cells. This was accompanied by an increase of proinflammatory cytokines and markers related to cancer progression. Hence we conclude that serglycin acts both as a modulating factor as well as one of the contributing factors in MC – glioma cell crosstalk. The present study provides evidence that serglycin can serve as a potential prognostic marker and has detrimental effects in MC infiltrated glioma microenvironment that requires further mechanistic investigations.

## MATERIALS AND METHODS

### Cell cultures and tissue microarrays

All cells were cultured at 37°C under 5% CO2. U2987MG, a human glioma cell line [41], was cultured in 10% FBS-containing MEM supplemented with 4mM L-glutamine, 100 units/ml penicillin and 0.1mg/ml streptomycin. The U2987MG cell line was established from a patient with high-grade glioma [41] and was denoted as primary culture 18. This cell line has then been very well studied [42, 43] and subsequently established as a stable glioma cell line. Other glioma cell cultures used in this study are derived from patients diagnosed with primary GBM and are part of the Uppsala University Human Glioma Cell Culture (HGCC) collection that comprises well characterized GBM-derived cell cultures [19]. U3017MG, U3084MG, U3086MG, U3039MG, U3042MG, U3035MG, U3071MG, U3078MG, U3054MG, U3047MG, U3117MG and U3019MG cells were cultured on laminin (10 mg/ml) in serum-free (stem cell) conditions, to enrich for stem-like glioma cells, as described previously [44].

The human MC line LAD2 (obtained from Prof Dean Metcalfe at NIH/NIAID, MD, USA) was cultured as described previously [45] in StemPro medium supplemented with 4 mM L-glutamine, 100units/ml penicillin and 0.1mg/ml streptomycin and 100ng/ml SCF (Invitrogen, Carlsbad, USA).

Tissue microarrays (TMAs) and slide scanning were performed using the strategies of The Human Protein Atlas project (www.proteinatlas.org) [46, 47]. TMAs involved tumor tissue from high-grade gliomas (anaplastic gliomas and glioblastomas, *n* = 101) and gliomas WHO grade II [25] (astrocytomas, oligoastrocytomas and oligodendrogliomas grade II, *n* = 87).

### Co-culture assays

To examine the effect of MCs on GBM cell growth and secretion, LAD2 cells were co-cultured in 6 well format transwells (0.4μm) with the various HGCC cell lines for 24, 36 and 48 hours. Briefly, the glioma cell lines were plated on laminin (10mg/ml) coated 6 well plates in serum-free (stem cell) conditions without growth factors and allowed to attach for 2–3 hours. For immunofluorescence staining laminin coated coverslips in 24 well plates were used for plating the glioma cells. Overnight SCF starved LAD2 cells were resuspended in normal growth medium without SCF (5 × 10^5^ cells/ml) and added to the transwell. The co-cultures were left to grow for 24, 36 and 48 hours. After the stipulated time-points the LAD2 cells were collected from the transwell and the cell-pellet was used for further analysis. The glioma cell lines were detached by Accutase. The cell pellet was washed in ice-cold PBS and then stored in −20°C and used for further analysis. When grown on coverslips, the glioma cells were washed with PBS followed by 3% PFA fixation for 10 min. Stimulation experiment was done in triplicates. Appropriate negative controls were kept for each experiment.

### RNA extraction, cDNA synthesis and qPCR

RNA was extracted from control LAD2 and control glioma cells as well as from LAD2 and glioma cells after co-culture. RNA extraction was done using GENEJET RNA Purification Kit (Life Technologies, Waltham, MA) extraction method from cell pellets. cDNA was synthesized using the High Capacity RNA-to-cDNA™ Kit (Thermo Fisher Scientific, Waltham, MA) which was then used to perform the qPCR using the PowerUp SYBR Mastermix (Table 1). For all qPCR analysis, ß-actin expression was used as endogenous control. Results are then calculated as increase in fold expression of the co-cultured cells compared to the expression levels without co-culture. The experiments were performed 3 times, with triplicates in each case.

**Table 1:** Human primers used for qPCR

Target gene	Forward sequence	Reverse sequence
Beta actin	GGACTTCGAGCAAGAGATGG	AGCACTGTGTTGGCGTACAG
ZEB1	GGCATACACCTACTCAACTACGG	TGGGCGGTGTAGAATCAGAGTC
SRGN	CGTCTGAGGACTGACCTTTTTCC	CGTTAGGAAGCCACTCCCAGAT
Vimentin	GTTTCCCCTAAACCGCTAGG	GACACGGACCTGGTGGAC
CD44	CCAGAAGGAACAGTGGTTTGGC	ACTGTCCTCTGGGCTTGGTGTT
MKI67	GAAAGAGTGGCAACCTGCCTTC	GCACCAAGTTTTACTACATCTGCC
CXCL10	GGTGAGAAGAGATGTCTGAATCC	GTCCATCCTTGGAAGCACTGCA
CXCL12	CTCAACACTCCAAACTGTGCCC	CTCCAGGTACTCCTGAATCCAC
TNF-α	CCCATGTTGTAGCAAACCCTC	TATCTCTCAGCTCCACGCCA
IL-6	GCCCTGAGAAAGGAGACAT	TTGTTTTCTGCCAGTGCCTC
CXCL1	AGCTTGCCTCAATCCTGCATCC	TCCTTCAGGAACAGCCACCAGT

### Immunohistochemistry and immunofluorescence

TMAs involved tumor tissue from high-grade gliomas (anaplastic gliomas and glioblastomas, *n* = 101) and gliomas WHO grade II [48] (astrocytomas, oligoastrocytomas and oligodendrogliomas grade II, *n* = 87). For immunohistochemical analysis, primary antibodies directed against human MC tryptase, hTPS (sc-33676, Santa Cruz Biotech, Santa Cruz, CA, USA), CD163 (HPA051974, Atlas Antibodies, Stockholm, Sweden), Serglycin (HPA000759, Atlas Antibodies, Stockholm, Sweden) and ZEB-1 (AMAb90510, Atlas Antibodies, Stockholm, Sweden) were used. Briefly, slides of tissue samples were deparaffinized in xylene, rehydrated in a series of aqueous solutions with decreasing concentrations of ethanol, boiled (125°C, 4 min) in epitope retrieval buffer (Thermo Scientific, Waltham, USA) in a pressure boiler and subsequently cooled for 30 min. Immunohistochemistry was then performed using the Autostained 480 instrument (Lab Vision, Freemont, USA) with 3′3′-diaminobenzidine (DAB) as a substrate. Thereafter, the slides were counterstained with hematoxylin, mounted and scanned using the ScanScope XT system (Aperio Technologies, Vista, USA).

Tissue microarrays preparation (TMAs), immunohistochemistry (IHC) and slide scanning were performed using the strategies of The Human Protein Atlas project (www.proteinatlas.org) [46, 47]

Immunofluorescent staining was carried out using primary antibodies against human CD3 (A0452, Dako, Agilent Technologies, CA USA), human CD8 (M7103, Dako, Agilent Technologies, CA USA) and Serglycin (sc-393521, Santa Cruz Biotech, Santa Cruz, CA, USA). Alexa 488 or Alexa 555 (Invitrogen, Carlsbad, USA) was used as secondary antibody. Briefly, deparaffinization was performed for the TMAs, whereas for staining of the cells a 10 minutes fixation in 4% PFA was performed. Then the slides were rinsed in PBS, blocked in 5% NGS in PBS-T (supplemented with 0.2% Triton-X 100 (Sigma Aldrich, St Loius, USA)) for 1 hour, followed by overnight incubation (4°C) with the primary antibody diluted in the blocking solution. The slides were subsequently incubated with appropriate secondary antibody for 45 min. Nuclei was stained with DAPI (1:5000) for 15 min and mounted in Immu-mount (Vecta Labs, Vector Labs, Burlingame, CA)

### Image analysis

Immunohistochemically stained TMA slides were scanned as described previously [47]. Slides with immunofluorescence staining were imaged using ZEISS AxioImager. Image analysis was done using ImageJ software.

### Bioinformatic analysis

Survival analysis was done using the TCGA GBM data downloaded from the cBio and TCGA portals. SERGLYCIN (SRGN) TCGA expression data (TCGA provisional, Affymetrix GeneChip Human Genome U133 Plus 2.0 Array, for 540 patients) [49] was downloaded from the cBio Portal (http://www.cbioportal.org/). Clinical data for these patients with primary GBM was obtained from the TCGA portal and in each case the maximum value of the survival times reported for a patient was used. Clinical data for comparing SRGN expression across different tumors was downloaded and statistically analyzed using the R2: Genomics Analysis and Visualization Platform (http://r2.amc.nl) and in each case datasets with large set of patient material was used. Transcriptome data for the HGCC collection was generated on Affymetrix GeneChip Human Exon 1.0 ST Arrays, RMA normalized and transformed to z-scores. HGCC cell lines were classified based on their expression profile for known signature genes [50] using known TCGA subtypes as reference and a k-nearest neighbour algorithm as described elsewhere [19].

### Statistical analysis

Statistical analyses were done using the Graphpad Prism software (GraphPad Software 6.0d) and R [51]. For group-wise comparisons the Student's unpaired *t*-test was used. For comparisons between more than two groups, one-way ANOVA tests were applied. Kaplan-Meier survival analysis was used for the TCGA and HGCC data and was performed with censoring at confidence interval 95%. The statistical significance of any observed survival difference was determined using the log-rank test. Correlation analyses employed Pearson coefficient analysis.

## SUPPLEMENTARY MATERIALS FIGURES AND TABLES


