# Supplementary material for: Serglycin as a potential biomarker for glioma: association of serglycin expression, extent of mast cell recruitment and glioblastoma progression
Source: Oncotarget. 2017 Mar 1;8(15):24815–27. doi: 10.18632/oncotarget.15820 (PMC5421891; doi:10.18632/oncotarget.15820)
Supplement: Supplementary file 1 [file oncotarget-08-24815-s001.pdf]

# Serglycin as a potential biomarker for glioma: association of serglycin expression, extent of mast cell recruitment and glioblastoma progression

## Supplementary Materials

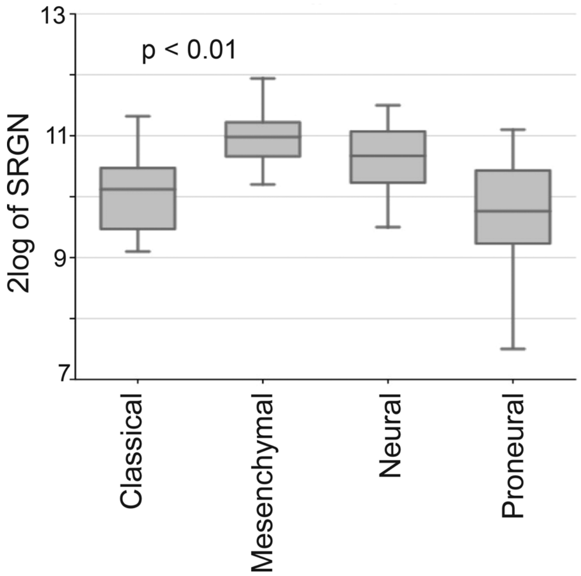


**Supplementary Figure 1: Expression levels of SRGN in the four GBM subtypes show significant variation.** Clinical data for the patients are from the TCGA portal and the analysis is done on the R2 genomic analysis platform. Difference in expression between groups was analysed using ANOVA. $p < 0.01$.

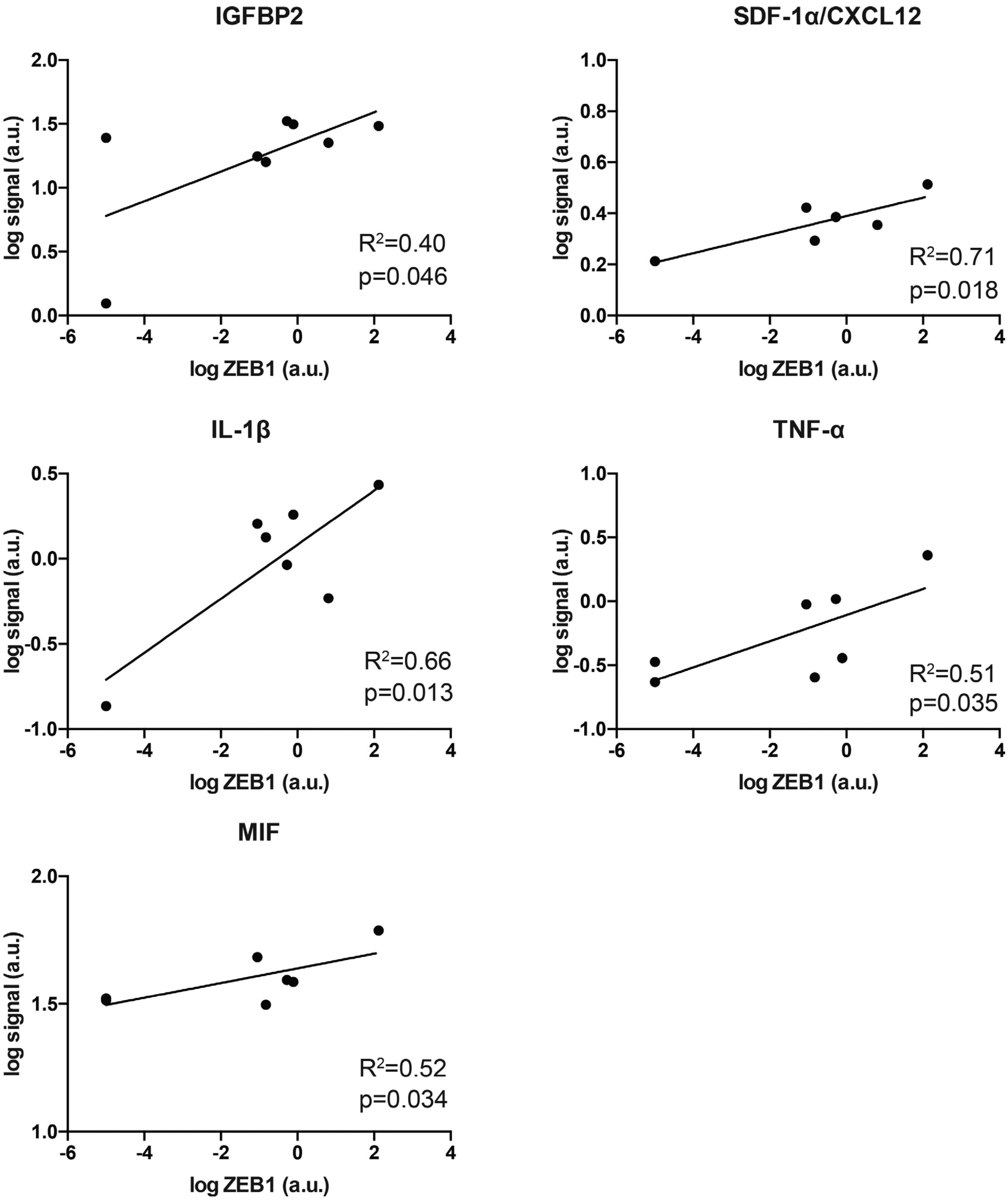


**Supplementary Figure 2: Analysis of GBM patient tumor specimens demonstrated that levels of pro-inflammatory cytokine SDF-1α (CXCL 12), IGFBP2, IL-1β, TNF-α and MIF correlate significantly with ZEB1.**

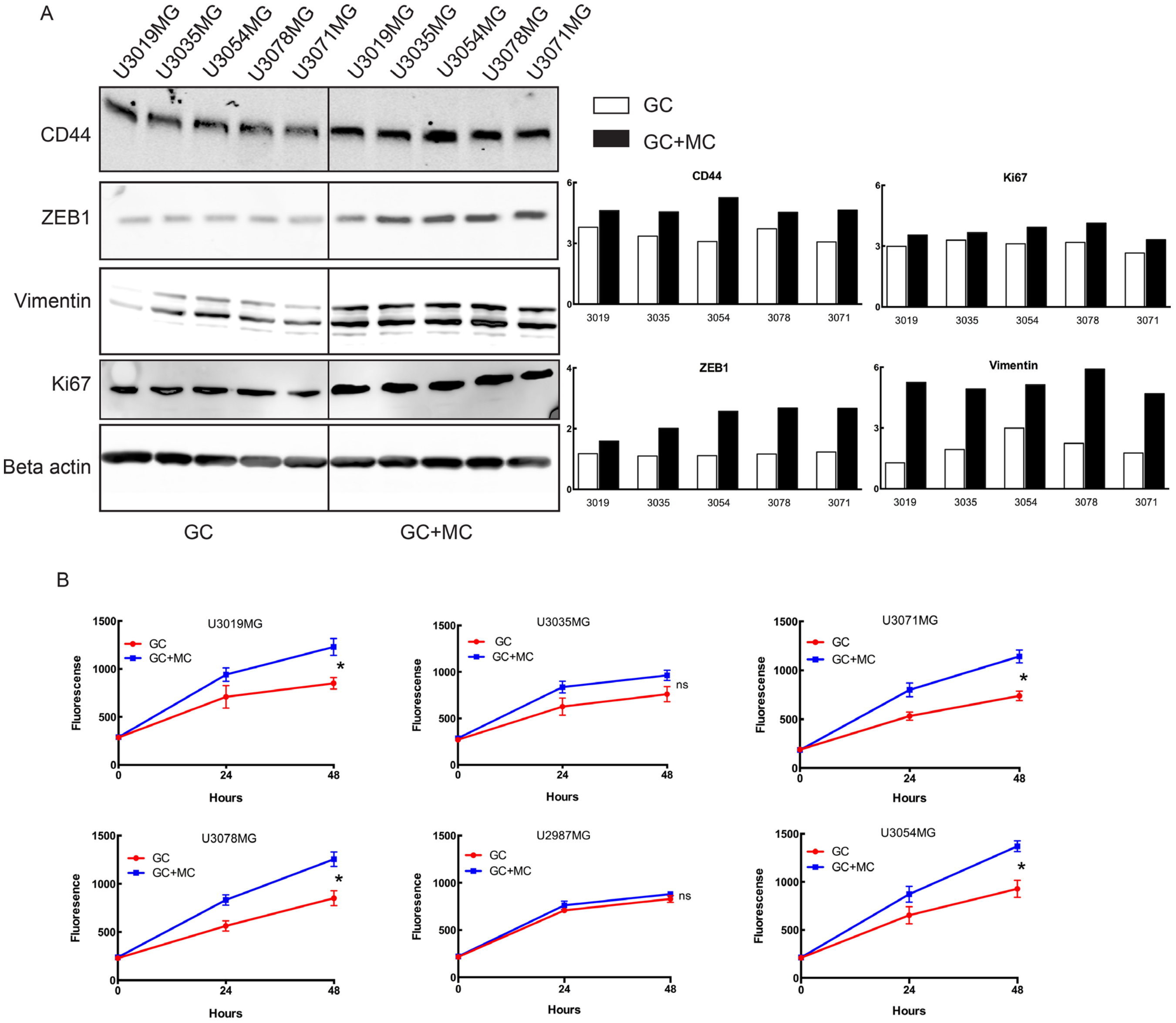


**Supplementary Figure 3:** (**A**) Western blot analysis showing expression levels of beta actin, Ki67, vimentin, ZEB1 and CD44 in the glioma cells before and after co-culture (**B**) CyQuant NF cell proliferation assay performed with the glioma cell lines grown alone or in co-culture with LAD2 cells. GC = glioma cells, GC + MC = glioma cells co-cultured with LAD2 cells. *$p < 0.05$.
